# Angiopoietin-1 receptor Tie2 distinguishes multipotent differentiation capability in bovine coccygeal nucleus pulposus cells

**DOI:** 10.1186/s13287-016-0337-9

**Published:** 2016-05-23

**Authors:** Adel Tekari, Samantha C. W. Chan, Daisuke Sakai, Sibylle Grad, Benjamin Gantenbein

**Affiliations:** Tissue and Organ Mechanobiology, Institute for Surgical Technology & Biomechanics, Medical Faculty, University of Bern, Bern, Switzerland; Biointerfaces, Empa, Swiss Federal Laboratories for Materials Science and Technology, St Gallen, Switzerland; Department for Orthopaedic Surgery, Tokai University School of Medicine, Isehara, Kanagawa Japan; AO Research Institute Davos, Davos, Switzerland; AO Spine Research Network, AO Spine International, Davos, Switzerland

**Keywords:** Intervertebral disc, Nucleus pulposus, Nucleus pulposus progenitor cells, Tie2, Hypoxia, Fibroblast growth factor 2, Growth factors

## Abstract

**Background:**

The intervertebral disc (IVD) has limited self-healing potential and disc repair strategies require an appropriate cell source such as progenitor cells that could regenerate the damaged cells and tissues. The objective of this study was to identify nucleus pulposus-derived progenitor cells (NPPC) and examine their potential in regenerative medicine in vitro.

**Methods:**

Nucleus pulposus cells (NPC) were obtained from 1-year-old bovine coccygeal discs by enzymatic digestion and were sorted for the angiopoietin-1 receptor Tie2. The obtained Tie2– and Tie2+ fractions of cells were differentiated into osteogenic, adipogenic, and chondrogenic lineages in vitro. Colony-forming units were prepared from both cell populations and the colonies formed were analyzed and quantified after 8 days of culture. In order to improve the preservation of the Tie2+ phenotype of NPPC in monolayer cultures, we tested a selection of growth factors known to have stimulating effects, cocultured NPPC with IVD tissue, and exposed them to hypoxic conditions (2 % O_2_).

**Results:**

After 3 weeks of differentiation culture, only the NPC that were positive for Tie2 were able to differentiate into osteocytes, adipocytes, and chondrocytes as characterized by calcium deposition (*p* < 0.0001), fat droplet formation (*p* < 0.0001), and glycosaminoglycan content (*p* = 0.0095 vs. Tie2– NPC), respectively. Sorted Tie2– and Tie2+ subpopulations of cells both formed colonies; however, the colonies formed from Tie2+ cells were spheroid in shape, whereas those from Tie2– cells were spread and fibroblastic. In addition, Tie2+ cells formed more colonies in 3D culture (*p* = 0.011) than Tie2– cells. During expansion, a fast decline in the fraction of Tie2+ cells was observed (*p* < 0.0001), which was partially reversed by low oxygen concentration (*p* = 0.0068) and supplementation of the culture with fibroblast growth factor 2 (FGF2) (*p* < 0.0001).

**Conclusions:**

Our results showed that the bovine nucleus pulposus contains NPPC that are Tie2+. These cells fulfilled formally progenitor criteria that were maintained in subsequent monolayer culture for up to 7 days by addition of FGF2 or hypoxic conditions. We propose that the nucleus pulposus represents a niche of precursor cells for regeneration of the IVD.

## Background

The intervertebral disc (IVD) has limited regenerative potential and disc degeneration is a major cause of chronic low back pain. This represents a leading cause of disability with significant economic and social burdens [1–3]. The IVD consists of an inner nucleus pulposus (NP) surrounded by the annulus fibrosus (AF) tissue, and hyaline articular cartilage is located at the endplates between the IVD and the vertebral bodies. The gelatinous NP is an avascular tissue containing a highly organized extracellular matrix rich in proteoglycans and collagens with few dispersed cells [4]. In this respect, the NP cells reside within hypoxic conditions, since no vasculature enters the NP [5]. Furthermore, disc cells actively regulate the homeostasis of the extracellular matrix by several cytokines and growth factors acting in an autocrine and paracrine fashion. Members of the transforming growth factor (TGF) superfamily, including TGFβ1, growth and differentiation factor, fibroblast growth factor 2 (FGF2), and vascular endothelial growth factor (VEGF) were identified previously as anabolic regulators within the IVD [6].

IVD degeneration implies a degradation of the extracellular matrix in the NP and the AF resulting in a reduced disc height. The exact mechanism by which IVD degeneration is induced is still unknown. Some risk factors were identified and include aging, genetic predisposition, and stress factors [7]. The degenerative changes of the IVD take place early in life and the cellular turnover rate is much slower compared with other tissues [8–10].

Current treatments aim to repair the degenerated disc by replacement of the injured tissue with a functional biological substitute or prosthesis. Conventional treatments for IVD degeneration are limited, since conservative or surgical therapies do not restore IVD tissue properties. Since the IVD possesses very limited healing capacity, regenerative medicine by injection of cells may represent promising therapy for treatment of disc degeneration [11]. As such, IVD repair strategies require an appropriate cell source that is able to regenerate the damaged NP tissue such as progenitor and stem cells. Cell-based therapies by injection of IVD cells, chondrocytes, or stem cells have gained significant insight and progressed to clinical trials for treatment of spinal disorders [12]. Progenitor cells do have the advantage over terminally differentiated cells that they maintain their multipotent differentiation and self-renewal potential in vivo and in vitro under appropriate conditions. Furthermore, these cells play an important role in the development and homeostasis of the IVD tissue. Recently, progenitor cells that are positive for the angiopoietin-1 receptor (Tie2) were identified in the mouse and human NP [13]. These cells, which express aggrecan and collagen type II, were shown to have progenitor-like multipotency. Tie2, also known as CD202b, is a cellular membrane receptor tyrosine kinase of the Tie family. This receptor contains immunoglobulin-like loops and an epidermal growth factor (EGF)-similar domain 2 [14]. Expressed mainly in endothelial cells, the angiopoietin groups of ligands, upon binding to their receptor Tie2, are known to regulate angiogenesis [15]. Tie2 signaling appears to be critical for endothelial smooth muscle communication and vascular maturation. Deletion of Tie2 or its ligand in transgenic mice is embryonic lethal and mice die from cardiac failure [16]. The contribution of Tie2 to IVD homeostasis, however, is still poorly understood. Here, we isolated primary nucleus pulposus cells (NPC) from bovine coccygeal discs and sorted these for the Tie2 marker, where the Tie2+ fraction of cells is suggested to represent the nucleus pulposus progenitor cells (NPPC) population. To demonstrate the stemness of the Tie2+ cells, we performed differentiation assays for the Tie2– and Tie2+ cell populations and then addressed their ability to form colonies in methylcellulose-based medium. Presence of these NPPC has never been demonstrated in bovine coccygeal IVD, a leading ex-vivo animal model for studying disc degeneration and regenerative approaches [17]. A second aim was to address the reported difficulties to maintain the phenotype of NPPC in culture [13] and to test different cell culture conditions to maintain and eventually expand these cells in vitro in monolayer culture.

## Methods

### NPC isolation

NPC were obtained from 1-year-old bovine tail discs within 4 hours post mortem (no ethical permit required) by sequential digestion of NP tissue with 1.9 mg/ml pronase (Roche, Basel, Switzerland) for 1 hour and 80 μg/ml collagenase II (260 U/mg; Worthington, London, UK) on a plate shaker at 37 °C overnight. The remaining undigested tissue debris was removed by filtration through a 100 μm cell strainer (Falcon, Becton Dickinson, Allschwil, Switzerland); subsequently the cell viability was determined by trypan blue exclusion. The isolated NPC were used for further analysis.

### Cell sorting and characterization by flow cytometry

**Fig. 1:**
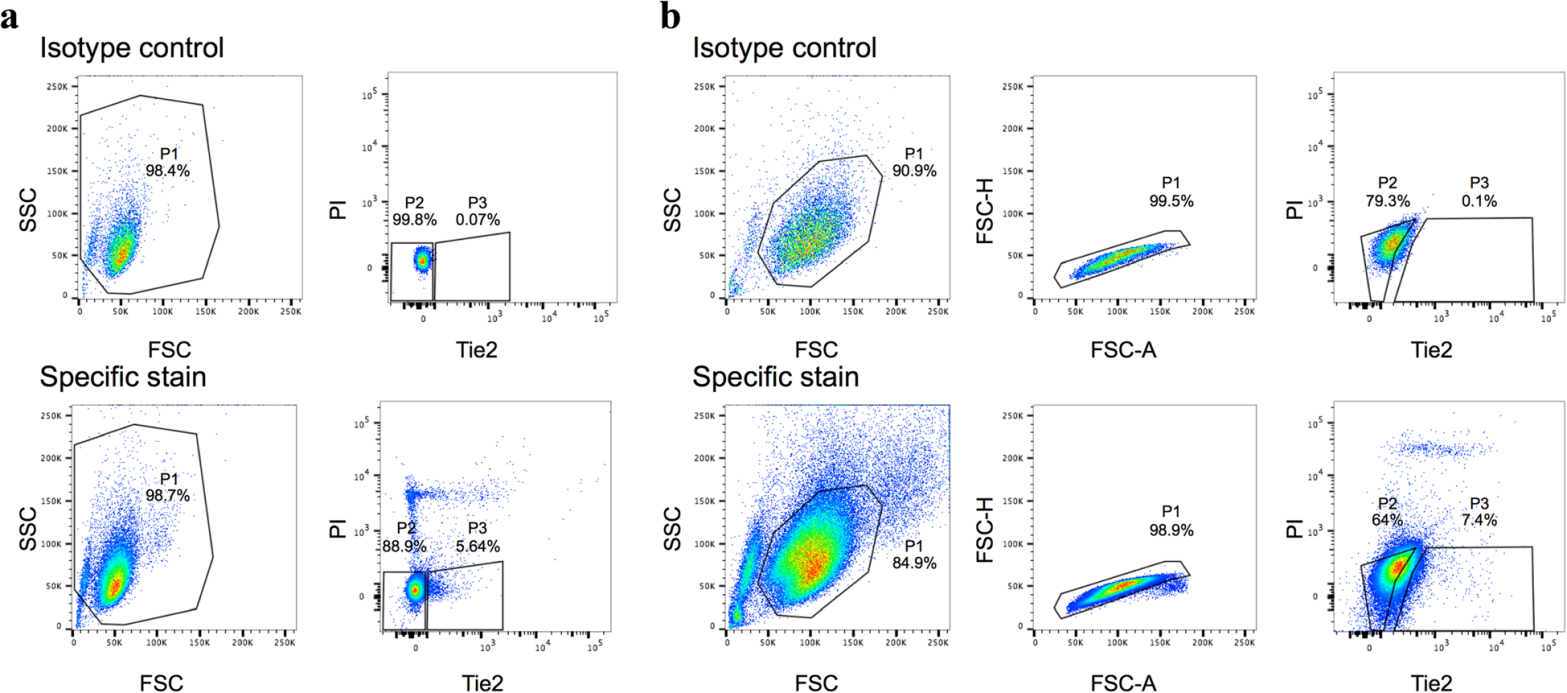
Sorting and gating strategies for Tie2+ cells from a whole NPC population. The NPC suspension after enzymatic digestion was colabeled with the Tie2 antibody and PI and sorted for the Tie2 marker. **a**, **b** Two examples show gating of the whole cell population for forward and side scatter (*FSC* and *SSC*, P1; *left panel*). It is important to mention that primary NPC after enzymatic digestion contain tissue fragments, granules of dead cells, and debris, which are removed by a selective gating for FSC and SSC (*left panel*, **b**). In addition doublets are excluded by a FSC-H versus FSC-A gating (*middle panel*, **b**). Proper gating for Tie2 is shown for the two examples and was performed by a negative selection of cells in isotype-matched control with less than 0.1 % (*top right panel*, P3) and setting the gate at the left for the Tie2– cells (P2). The same gating was then applied for the specific Tie2 staining and by excluding PI-positive cells. *P1* whole NPC population, *P2* Tie2– cell population, *P3* Tie2+ cell population, *PI* propidium iodide, *Tie2* angiopoietin-1 receptor

To isolate the fraction of Tie2 expressing cells, NPC were labeled as described previously [13]. Briefly, the NPC population obtained after enzymatic digestion of 6-8 IVDs (about 8 × 10^6^ cells for one bovine tail) was resuspended in 100 μl of fluorescence-activated cell sorting (FACS) buffer (phosphate-buffered saline containing 0.5 % bovine serum albumin (Sigma-Aldrich, Buchs, Switzerland) and 1 mM EDTA (Fluka, Buchs, Switzerland)) and was incubated with anti-rat Tie2/CD202b polyclonal rabbit antibody (10 μg/ml, clone bs-1300R; Bioss Antibodies, Woburn, MA, USA) for 30 min at 4 °C. Incubation was performed for a further 30 min at 4 °C with goat anti-rabbit antibody (Molecular Probes, Life Technologies, Zug, Switzerland) labeled with the fluorochrome Alexa 488. Isotype-matched antibody (Invitrogen, Life Technologies) was used as negative control to set the appropriate gate for positive Tie2 cells (Fig. 1). Sorting was performed on FACS Diva III (BD Biosciences, San Diego, USA); only living cells were considered by using the propidium iodide (PI)-negative gate.

To characterize the NPC by Tie2 expression after expansion in monolayer culture, the cells were labeled in a similar way. Briefly, 2 × 10^5^ NPC in 100 μl of FACS buffer were stained with the anti-rat Tie/CD202b antibody for 30 min at 4 °C and further incubated with the goat anti-rabbit secondary antibody for 30 min at 4 °C. Fluorescence was measured on an LSR II flow cytometry system (Becton Dickinson), and the data were analyzed using FlowJo software (version 10.1 for MacOS X; LLC, Ashland, OR, USA).

### NPPC proliferation

To identify proliferating cells, NPPC were expanded for 7 days in proliferation medium (alpha minimum essential medium (α-MEM; Gibco, Life Technologies) containing 10 % fetal bovine serum (FBS; Sigma-Aldrich) and penicillin/streptomycin (P/S, 100 units/ml and 100 μg/ml, respectively; Merck, Darmstadt, Germany)), whereby 10 μM bromodeoxyuridine (BrdU) was added at the beginning of the experiment with one medium change. The incorporated BrdU was detected by flow cytometry according to manufacturer’s instructions (APC BrdU Flow Kit; Becton Dickinson).

### Colony-forming assay

To assess the formation of colonies, single-cell suspensions of 10^3^ NPC were seeded in 1 ml of methylcellulose-based medium (MethoCult H4230; Stem Cell Technologies, Vancouver, Canada) in Petri dishes (35 mm in diameter) and cultured for 8 days. The colonies formed (>10 nuclei) were quantified under a light microscope.

### Osteogenic differentiation

Differentiation of NPC into osteogenic lineage was performed for cells immediately after digestion of the NP and sorting for Tie2, and was conducted in α-MEM containing 5 % FBS, P/S, 100 nM dexamethasone, 10 mM β-glycerophosphate, and 0.1 mM l-ascorbic acid-2-phosphate (all from Sigma-Aldrich) for 21 days with medium change twice a week. The serum concentration was chosen according to a pilot study (data not shown) showing a better differentiation of NPPC into osteogenic lineage during the given time period. To evaluate the cells’ ability for calcium deposition, Alizarin red staining was performed. The cell layers were fixed in 4 % formaldehyde, rinsed with distilled water, and subsequently exposed to 2 % Alizarin red solution for 45 min. The Alizarin red staining was released from the cell layers by addition of 10 % cetylpyridinium chloride solution (Sigma-Aldrich) and incubation for 1 hour with vigorous agitation. The samples were diluted 10-fold, transferred into a 96-well plate, and the optical density was measured at 570 nm using a microplate reader (SpectraMax M5; Bucher Biotec, Basel, Switzerland).

### Adipogenic differentiation

Immediately after digestion of the NP and sorting for Tie2, NPC were grown in adipogenic medium consisting of α-MEM with 5 % FBS, P/S, 12.5 μM insulin, 100 nM dexamethasone, 0.5 mM isobutylmethylxanthine, and 60 μM indomethacin (all from Sigma-Aldrich) with medium change twice a week. Adipogenic differentiation was evaluated after 3 weeks of induction by the cellular accumulation of lipid vacuoles that were stained with Oil red O (Merck). The cell layers were fixed in 4 % formaldehyde, rinsed with 50 % ethanol, subsequently stained with Oil red O solution for 20 min, and counterstained with Mayer’s Hematoxylin (Fluka) for 3 min. The cellular accumulation of lipids was quantified from the wells by counting the Oil red O-positive cells under a light microscope.

### Chondrogenic differentiation

The NPC were expanded in proliferation medium in 6-well plates to compensate for the low number of Tie2+ cells obtained after sorting. Near confluency (1.93 ± 0.32 (mean ± SD) population doublings), the NPC were resorted and the different NPC populations (Tie2–, Tie2+, and unsorted NPC) were induced towards chondrogenic differentiation. Briefly, 2.5 × 10^5^ cells in Dulbecco’s modified Eagle’s medium–high glucose (with 4.5 g/l glucose; Gibco) containing P/S, ITS+, 0.1 mM L- ascorbic acid-2-phosphate, 0.3 mM l-proline, 100 nM dexamethasone (all from Sigma-Aldrich), and 10 ng/ml TGFβ1 (Peprotech, London, UK) were transferred into 15 ml polypropylene tubes and centrifuged at 500 × *g* for 5 min [18]. After 3 weeks of culture, the pellet cultures were fixed with 4 % formaldehyde solution for 4 hours at room temperature and embedded in paraffin for subsequent preparation of 5 μm-thick sections. Sulfated glycosaminoglycans (GAG) were stained with 0.2 % Safranin-O for 10 min and sections counterstained with 0.04 % Fast Green for 2 min.

To quantify the GAG content, the pellets were recovered by melting the paraffin blocks and subsequently digested with a 3.9 U/ml papain solution containing 5 mM sodium citrate, 150 mM cysteine hydrochloride, and 5 mM EDTA (Sigma-Aldrich) at 60 °C overnight. The total GAG content was quantified from the lysates using a bovine cartilage chondroitin sulfate standard (Sigma-Aldrich) and normalized to the DNA content (Picogreen ds DNA Assay kit; Molecular Probes, Life Technologies).

Immunohistochemical staining for proteoglycans was performed by incubation of the sections with a monoclonal mouse anti-human proteoglycan antibody (10 μg/ml, clone EFG-4; Millipore, Billerica, MA, USA) at 4 °C overnight after permeabilization with 100 % methanol for 2 min and blocking with 10 % FBS for 1 hour. Incubation was performed for a further 1 hour with a goat anti-mouse secondary antibody (Alexa 488; Molecular Probes, Life Technologies). The tissues were visualized with a confocal laser-scanning microscope (cLSM 710; Carl Zeiss, Jena, Germany).

### Expansion of Tie2+ cells and culture conditions

The freshly isolated Tie2+ cells after sorting were treated with various growth factors and oxygen concentrations to test for culture conditions that could amplify and maintain the Tie2+ cells. Growth factors (Peprotech), including growth differentiation factor 5 (GDF5), GDF6, EGF, VEGF, FGF2 (100 ng/ml), and TGFβ1 (10 ng/ml), or coculture with IVD tissue using culture inserts (Becton Dickinson) for 6-well plates were applied to Tie2+ cells after sorting for 7 days in normoxia. The concentrations of the growth factors were selected according to previously published results showing a beneficial effect on NPC and/or maintenance and proliferation of stem cells in vitro [19–25]. Hypoxic conditions at 2 % O_2_ have been shown in multiple studies [26, 27], including by our group [19, 28], to have a stimulatory effect on aggrecan expression by NPC. To test for cell proliferation and the conservation of Tie2 markers under hypoxia, Tie2– and Tie2+ cells were cultured in normoxia (atmospheric O_2_, ~21 %) or in hypoxia using a C-274-2 shelf chamber inside a standard incubator and 1× Pro-Ox controller (Biospherix, Union Street Parish, New York, USA) adjusted to 2 % O_2_ by addition of N_2_.

### Real-time RT-PCR

Relative gene expression of Tie2 (*TEK*), collagen type II (*COL2*), aggrecan (*ACAN*), hypoxia-inducible factor 1 alpha (*HIF1α*), and ribosomal *18S* RNA as a reference gene were monitored on expanded NPC. In order to determine the baseline expression levels of selected genes, bovine-specific oligonucleotide primers (Table 1) (Microsynth, Balgach, Switzerland) were newly designed with Beacon Designer™ software (Premier Biosoft, Palo Alto, CA, USA) based on nucleotide sequences from GenBank. All primers were tested for efficiency and melting curves of amplicons were performed to determine specific amplification. Relative gene expression was determined by application of a threshold cycle and normalization to the reference sample (primary Tie2– NPC on day 0) using the 2^–ΔΔCt^ method according to Livak and Schmitten [29].

**Table 1:** Custom-designed DNA primers used in real-time quantitative PCR study

Gene	Forward sequence	Reverse sequence
*18S*	ACGGACAGGATTGACAGATTG	CCAGAGTCTCGTTCGTTATCG
*TEK*	GGACAGGCAATAAGGATACG	ACCGAGTGGATGAAGGAA
*COL2*	CGGGTGAACGTGGAGAGACA	GTCCAGGGTTGCCATTGGAG
*ACAN*	GGCATCGTGTTCCATTACAG	ACTCGTCCTTGTCTCCATAG
*HIF1α*	AGGTGGATATGTCTGGATA	CAAGTCGTGCTGAATAATAC

Amplicons were generated using a two-step amplification cycling (95 °C for 15 s and 57 °C for 30 s for 45 cycles) and SYBR-green mastermix

TEK angiopoietin-1 receptor gene, COL2 collagen type II gene, ACAN aggrecan gene, HIF1α hypoxia-inducible factor 1 alpha gene, 18S ribosomal 18S RNA

### Statistical analysis

Differences in the number of colonies (*N* = 6 animals), BrdU-positive cells (*N* = 3), and expression of Tie2 (*N* = 3) were evaluated by Student’s *t* test; histological quantifications (*N* = 5), levels of transcripts (*N* = 5), and Tie2+ cell fractions (*N* = 3) were evaluated by one-way ANOVA with Bonferroni’s post-hoc test, using GraphPad Prism (version 6.0 h for Mac OS; GraphPad Software Inc., La Jolla, CA USA). *p* < 0.05 was considered significant.

## Results

### Sorting of Tie2+ cells from isolated NPC

The fraction of sorted Tie2+ cells after isolation of NPC accounted for 8.66 ± 3.94 % (values presented as mean ± SD) of the entire NPC population (*N* = 10 animals). The amount of Tie2+ cells showed slight variation (variation coefficient = 45.6 %) among the donors.

### Differentiation of NPC in vitro

**Fig. 2:**
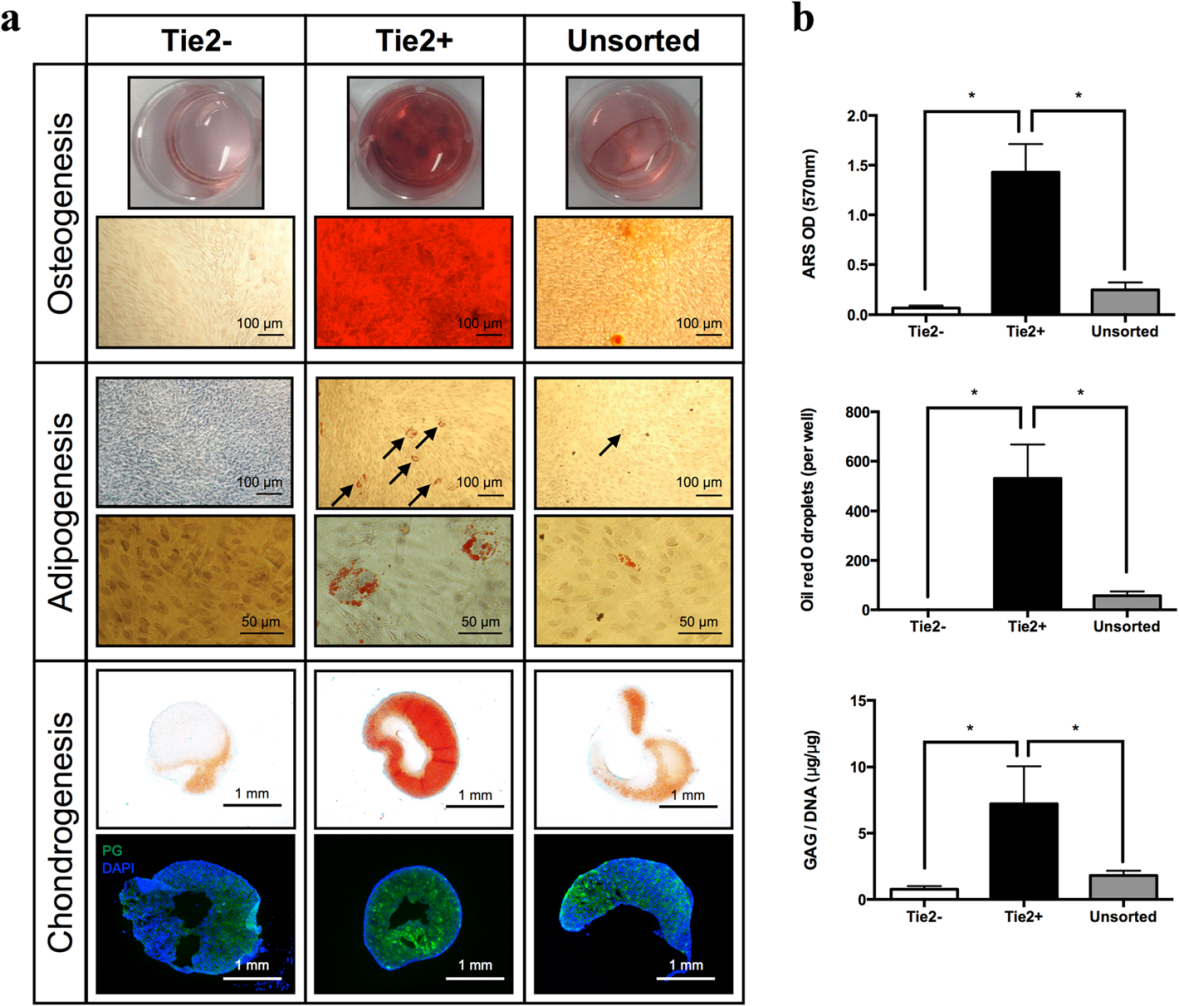
Osteogenic, adipogenic, and chondrogenic differentiation assays. **a** Differentiation assays were performed in Tie2– cells and Tie2+ cells (i.e., NPPC) after sorting and a mixed cell population (unsorted NPC). *Top panel* Macroscopic and microscopic images of osteogenesis (Alizarin red staining). *Middle panel* Adipogenic differentiation (Oil red O staining), *arrows* highlighting the formation of fat droplets. *Lower panel* Chondrogenic differentiation: Safranin-O staining and proteoglycans (*PG*, *green*) immunohistochemistry counterstained with 4′,6-diamidino-2-phenylindole (*DAPI*, *blue*). Results of one representative experiment of at least three repeats are shown. Scale bars are indicated on the images. **b** Quantification of Alizarin red staining (*ARS*), Oil red O fat droplet-positive cells, and *GAG*/DNA content. Individual cell populations were cross-compared to determine significance with *p < 0.05. Bars represent mean ± SD (*N* = 5). *GAG* glycosaminoglycans, *Tie2* angiopoietin-1 receptor (Color figure online)

For the differentiation assays of NPC into osteogenic, adipogenic, and chondrogenic lineages, we considered the sorted Tie2– cells, the sorted Tie2+ cells, and a mixed NP population of cells (unsorted) for comparison. After 3 weeks of osteogenic induction, the cell layer formed with Tie2– cells was negative for Alizarin red and no calcium deposition was observed (Fig. 2). By contrast, Tie2+ cells deposited an extensive mineralized matrix in osteogenic medium, as demonstrated by strong Alizarin red staining (*p* < 0.0001). Interestingly, some mineralized nodular formation was observed with a mixed cell population; however, the amount of Alizarin red staining did not significantly differ (*p* = 0.37) from Tie2– cells. The adipogenic differentiation of NPC showed that Tie2– cells could not form adipocytes; however, cellular accumulation of lipid vacuoles was detected within the Tie2+ cells as demonstrated by a positive staining with Oil red O. The number of Oil red O-positive cells was significantly higher in Tie2+ cells (*p* < 0.0001) as compared with Tie2– cells. Some fat droplets were detected within the culture of unsorted cells but to a lesser extent compared with Tie2+ cells (*p* < 0.001). However, this did not significantly differ from Tie2– cells (*p* = 0.85). For chondrogenic differentiation, the tissue formed with Tie2– cells stained very weakly for GAG (by Safranin-O) and the cells showed a fibroblastic morphology. However, the cultures with Tie2+ cells stained intensely for GAG with lacunae formation observed, a characteristic of a cartilaginous phenotype, and a higher GAG/DNA content (*p* = 0.0095) compared with Tie2– cells. Similarly, the unsorted cells were able to form a cartilage-like tissue, although staining was less intense compared with the tissue of Tie2+ cells (*p* = 0.02). Similar results were observed for the proteoglycan immunohistochemistry staining, where the highest amount was detected within tissue formed from Tie2+ cells and lower amounts were observed for unsorted and Tie2– cells.

### Colony formation

**Fig. 3:**
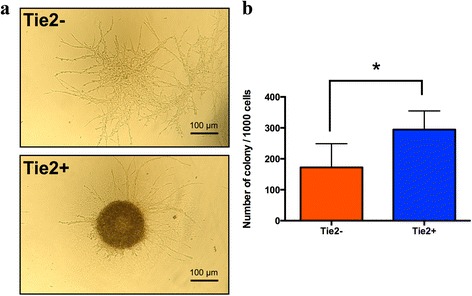
Colony-forming assay of NPC versus NPPC. **a** Macroscopic images and (**b**) quantification of colonies (>10 cells) formed in Tie2– and Tie2+ cells after 8 days of culture in methylcellulose-based medium (*N* = 6). *p < 0.05 compared with Tie2– colonies. *Tie2* angiopoietin-1 receptor

The Tie2– and Tie2+ isolated cell populations were able to form colonies after 8 days of culture in methylcellulose-based medium. However, the colonies formed with Tie2– cells were spread, plastic adherent, and fibroblastic, whereas the Tie2+ colonies formed were spheroid and rounded as observed macroscopically (Fig. 3a). The colonies of Tie2+ cells were quantitatively more abundant (*p* = 0.011) compared with Tie2– colonies (Fig. 3b).

### Proliferation of Tie2+ cells in monolayer cultures

**Fig. 4:**
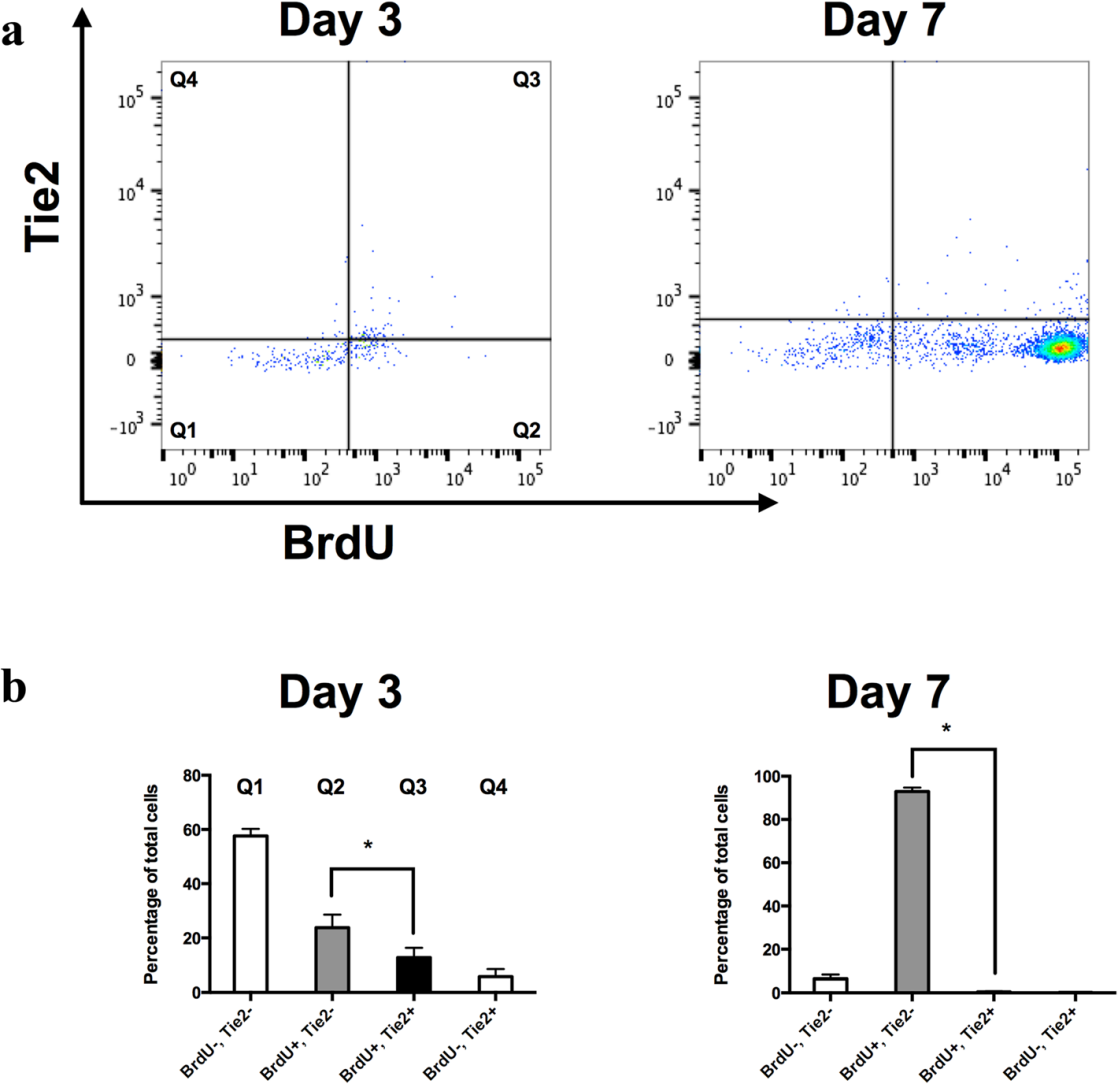
Proliferation of NPPC. Primary NPPC were labeled with BrdU for 3 and 7 days before the end of culture. **a** Incorporated BrdU, in combination with surface-bound Tie2, was assessed using flow cytometry. **b** Proportion of each cell population determined from the scatter plot quartiles (*N* = 3). *p < 0.05 compared with Tie2– cells. *BrdU* bromodeoxyuridine, *Tie2* angiopoietin-1 receptor

After 3 days of culture, 18.49 ± 4.30 % of the NPPC were positive for Tie2 (Fig. 4), while this fraction dropped to 0.61 ± 0.31 % after 7 days. The fraction of BrdU-positive cells increased from 36.56 ± 1.01 % to 93.36 ± 1.56 % when the cells were exposed to BrdU for 3–7 days. The fraction of Tie2+ cells showed a higher proliferative capacity on day 3 compared with Tie2– cells (69.2 ± 8.26 % vs. 29.1 ± 8.26 %, values defined as the ratio of BrdU-positive cells of total Tie2– or Tie2+ cells), while Tie2+ cells were less proliferative on day 7 (64.3 ± 13.4 % vs. 93.5 ± 1.52 %). Cells that incorporated BrdU were found to be either Tie2+ or Tie2–.

### Expression of Tie2 during expansion of NPC

**Fig. 5:**
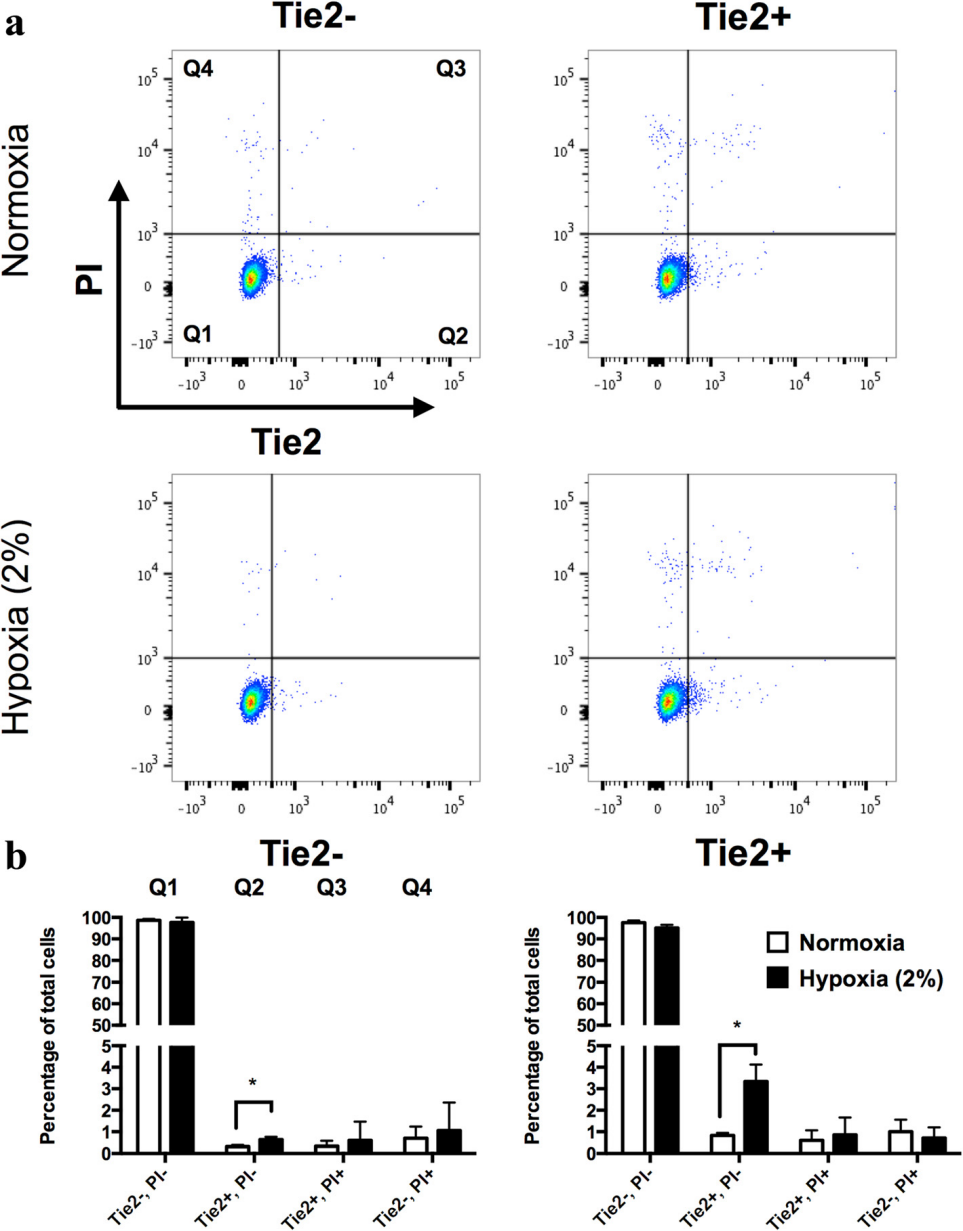
Expression levels of the surface-bound Tie2. **a** Expression levels of Tie2 after expansion for 7 days in monolayer cultures of Tie2– and Tie2+ cell (NPPC) populations in normoxic and hypoxic conditions were assessed using flow cytometry. **b** Quantification from the different scatter plot quartiles. The NPC were costained with PI. *p < 0.05 compared with normoxia. Values are mean ± SD compared with the isotype control (*N* = 3). *PI* propidium iodide, *Tie2* angiopoietin-1 receptor

The expression of Tie2 was monitored during expansion of primary NPC in monolayer cultures. Therefore, Tie2– and Tie2+ cells after sorting were plated in 6-well plates at a density of 3 × 10^4^ cells/well and kept in the proliferation medium for 7 days in a normoxia or hypoxia environment (2 % O_2_). The cells were harvested and processed for flow cytometry analysis by staining for the Tie2 marker. It was found that the fraction of Tie2+ cells was rapidly lost in monolayer cultures in both normoxic and hypoxic conditions (Fig. 5), although culture of the NPPC in hypoxic conditions better maintained the Tie2+ pool of cells (3.34 ± 0.78 %) compared with normoxia (0.83 ± 0.12 %). The proportion of Tie2+ cells of the expanded Tie2– cells was nearly absent after 7 days of culture, which accounted for 0.31 ± 0.08 % in normoxia and 0.63 ± 0.14 % in hypoxic conditions. More than 95 % of the cells were viable in both culture conditions as detected by negative PI staining.

### Gene expression

**Fig. 6:**
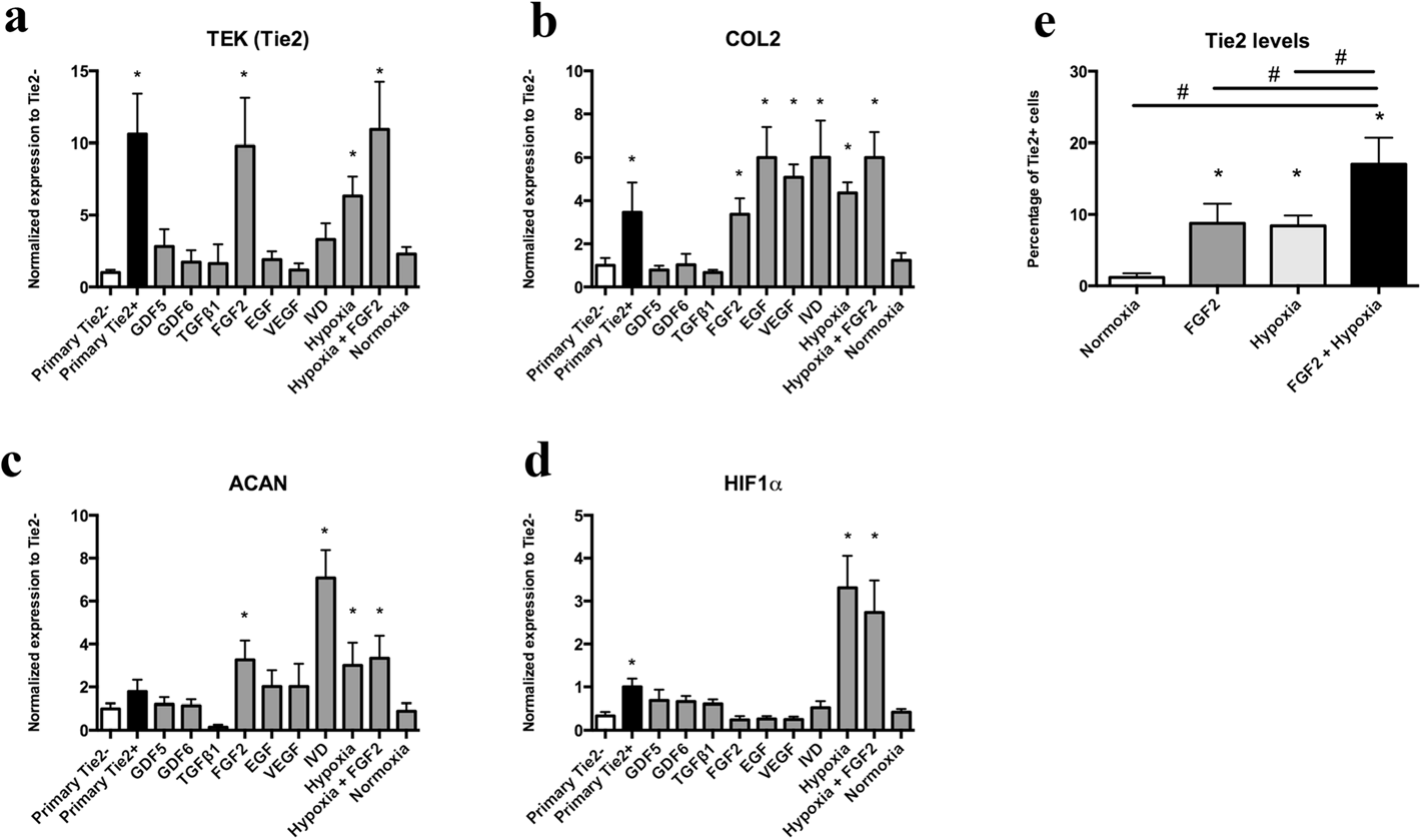
Maintenance of NPPC (Tie2+) phenotype. Primary NPPC were stimulated with various growth factors (growth and differentiation factor 5 (GDF5), GDF6, transforming growth factor β1 (TGFβ1), fibroblast growth factor 2 (FGF2), epidermal growth factor (EGF), and vascular endothelial growth factor (VEGF)), cocultured with IVD tissue or subjected to hypoxic conditions for 7 days, and the transcript levels of Tie2 were measured: endothelial tyrosine kinase (*TEK*) (**a**), collagen type 2 (*COL2*) (**b**), aggrecan (*ACAN*) (**c**), and hypoxia-inducible factor 1 alpha (*HIF1α*) (**d**). Values are mean ± SD (*N* = 5). *p < 0.05 compared with Tie2– of primary NPC. Protein level of Tie2 was monitored by flow cytometry in NPPC that were subjected to hypoxic conditions and/or FGF2 (**e**). Values are mean ± SD (*N* = 3). *p < 0.05 compared with normoxic conditions. #*p* < 0.05 compared with FGF2 + hypoxic conditions. *IVD* intervertebral disc, *Tie2* angiopoietin-1 receptor

The isolated Tie2+ NPPC were cultured in the proliferation medium in the presence of various growth factors or cocultured with IVD tissue for 7 days in normoxic conditions. Alternatively, cells were cultured under hypoxic conditions with/without FGF2. Treatment of the cells with FGF2 (100 ng/ml) and/or culture under hypoxic conditions resulted in a significant increase of *TEK* gene expression to levels comparable with Tie2+ after sorting (Fig. 6a). FGF2, EGF, VEGF (100 ng/ml), coculture with IVD tissue, and hypoxia increased collagen type 2 (Fig. 6b) and aggrecan expression (Fig. 6c) compared with Tie2– after sorting or cultures of NPPC for 7 days in normoxia without growth factor or IVD tissue. No such effect was detected when NPPC were treated with GDF5, GDF6 (100 ng/ml), or TGFβ1 (10 ng/ml). *HIF1α* was significantly increased in hypoxic conditions (Fig. 6d). A synergistic effect of FGF2 and hypoxia on the transcript (Fig. 6a) and protein levels (Fig. 6e) of Tie2 was observed.

## Discussion

Cell-based treatment of disc degeneration represents a promising approach to restore the IVD tissue function and to relieve pain [30–32]. Extensive research in the past decade using different animal models and clinical trials has improved our knowledge on the effects of cell-based therapies. In these studies, different cell types including IVD-derived cells [33–35], chondrocytes [36–38], and stem and progenitor cells [39–43] were used for transplantation into the degenerated IVD either alone or in combination with a biomaterial. The success rate of such treatments was variable and highly dependent on the model used, indicating that the selection of the cell source is a crucial parameter for treatment of disc degeneration. Bone marrow or adipose tissue-derived stem and progenitor cells might have the advantage over committed cells in that they can be isolated in large quantities and without donor site morbidity. Importantly, these cells possess multipotent properties and have a proliferative capacity, which make these cells attractive for delivery into degenerated discs. Preclinical studies showed that cells from the mesenchymal origin can participate in disc regeneration by differentiating into chondrocyte-like cells and producing NP tissue-specific extracellular matrix, namely aggrecan and collagen type 2. Because NPC share some similarities in phenotype and molecular content with cartilage-specific cells, the chondrocytes [44], those cells with the ability to differentiate into chondrocytes are considered a potential target for the regeneration of the IVD tissue. Resident progenitor cells within the IVD were documented previously [45–47]. Some of these cells were shown to maintain multipotent and self-renewal potential when cultured in vitro; however, little is known about their role in the homeostasis of the IVD.

Within this study, we demonstrated that Tie2+ cells from the bovine coccygeal discs are progenitor-like/multipotent cells, which are able to differentiate into osteogenic, adipogenic, and chondrogenic lineages in vitro. Sakai et al. [13] were the first to identify NP progenitor cells in the Tie2+ and disialoganglioside 2 positive (GD2+) cell fraction from human and mouse IVD tissues. These cells were described to derive from the Tie2+ and GD2– precursor cells and are capable of differentiating into multiple mesenchymal and NP lineages. GD2 was described as an additional marker for progeny, whose expression is increased with activation and commitment of the NP progenitor cells. In this study, the expression of GD2 and its contribution to differentiation of the disc cells was not investigated. Our findings show the presence of Tie2+ NPPC in the bovine coccygeal discs and further support previous results on NPPC in human and in mice [13, 48]. Additionally, we showed that in contrast to Tie2–, only Tie2+ cells have a multipotent potential as characterized by their differentiation capacity in vitro and their ability to form spheroid colonies. Tie2– cells within the disc tissue could therefore be considered NP committed cells. The NPPC may represent the key cells for the regenerative capacity of the disc and maintenance of these cells could contribute to the homeostasis of the IVD.

NPPC were first described from human and mouse IVDs [13]. Here, we could successfully isolate them from bovine coccygeal IVDs. These IVDs have been established as a reliable model to assess the biology and biomechanics of the disc [49–51]. The present findings allow further investigations and subsequent translation into human samples, which are clinically more relevant.

Applying flow cytometry to detect the surface-bound Tie2 marker allowed us to investigate the two phenotypes present within a pool of expanded NPC. It should be noted that setting the appropriate gate for Tie2 during sorting of the NPC is highly sensitive and should be made very stringent in order to avoid isolation of Tie2– cells, which may not demonstrate multipotent differentiation potential. In primary NPC, 8.66 ± 3.94 % of the cells stained positive for Tie2. During expansion, the proportion of Tie2+ cells was rapidly lost in subsequent monolayer cultures and less than 1 % could be detected after 2.31 ± 0.28 (mean ± SD) population doublings. In support of our data are studies investigating molecular changes of progenitor cells during in vitro monolayer cultures, where they found that cellular morphology, self-renewal, and differentiation capacity of these cells are altered during expansion [52–54].

When the primary NPC were subjected to monolayer cultures, they adhered and started to proliferate within a few days. Because 8.66 ± 3.94 % of the freshly isolated NPC population expressed Tie2, we wondered whether the proliferating pool of cells comprised Tie2+ cells or whether this pool is restricted to Tie2– cells. The present data confirmed that proliferating cells showed both Tie2+ and Tie2– phenotypes. These experiments showed that cells harvested from the NP tissue are able to maintain, at least for a short period, synthesis of Tie2 while proliferating in monolayer cultures. Furthermore, we found that Tie2+ cells have a higher proliferative capacity after 3 days compared with the Tie2– cell fraction, while the Tie2+ fraction showed less proliferative activity on day 7. The proliferation dynamics of Tie2+ cells could therefore be explained by the massive increase of the Tie2– pool of cells and the loss of the Tie2+ fraction during expansion.

We addressed protocols for enrichment of Tie2+ cells in vitro by application of growth factors known to be beneficial for NPC or for inducing angiogenesis, by varying oxygen concentrations, or by coculture with IVD tissue. Supplementation of the cultures with FGF2 increased the TEK expression to levels similar to primary Tie2+ NPC. FGF2 is known as a potent inducer of angiogenesis [55] and was described as a crucial factor for the successful maintenance of the undifferentiated state and self-renewal of stem cells. Lotz et al. [56] reported that stabilization of FGF2 using controlled poly(lactic-co-glycolic acid) (PLGA) microsphere delivery improves the expression of stem cell markers and cell amplification, and decreases spontaneous differentiation. In addition to FGF2, low oxygen concentrations (2 % O_2_) better maintained the Tie2+ pool of cells compared with normoxia; while simultaneous supplementation of the cultures with FGF2 and hypoxic conditions showed a synergistic effect and better maintained the Tie2 expression in NPPC after 7 days of culture. Physiological hypoxic conditions were previously suggested to maintain the undifferentiated state of many precursor cells, including embryonic, hematopoietic, mesenchymal, and neural stem cells [57]. Furthermore, cells of the IVD reside within a hypoxic environment and are preserved throughout their lifespan.

To characterize the NPPC during expansion, and following supplementation with various growth factors and coculture with IVD tissue, we performed a gene expression analysis of two key genes for the NP, namely aggrecan and collagen type 2. It was found that VEGF, EGF, FGF2, or coculture with IVD increased the expression of NP markers, suggesting the contribution of these factors to differentiation of the NPPC towards the NP phenotype. Surprisingly, exposure of NPPC to recombinant GDF5, GDF6, and TGFβ1 could not increase the expression of aggrecan or collagen type 2. An explanation may derive from the fact that these factors might be active in committed NPC rather than in progenitor cells. These growth factors were shown previously to enhance the discogenic phenotype of bone marrow-derived mesenchymal stromal cells in vitro [21] while a stage-dependent TGFβ1-induced chondrogenic differentiation of embryonic stem cells was observed [58].

## Conclusions

The data presented herein demonstrate the presence of a progenitor cell population within the NP expressing the cell surface marker Tie2 and being able to differentiate into osteogenic, adipogenic, and chondrogenic lineages in vitro. Strategies to maintain the Tie2+ pool of the NPC merit further evaluation, and sorting for Tie2 may contribute to a more suitable source for cell therapy for regeneration of the IVD.

## Abbreviations

AF: annulus fibrosus
α-MEM: alpha minimum essential medium
BrdU: bromodeoxyuridine
EGF: epidermal growth factor
FACS: fluorescence-activated cell sorting
FBS: fetal bovine serum
FGF2: fibroblast growth factor 2
GAG: glycosaminoglycans
GD2: disialoganglioside 2
GDF: growth differentiation factor
HIF1α: hypoxia-inducible factor 1-alpha
IVD: intervertebral disc
NP: nucleus pulposus
NPC: nucleus pulposus cells
NPPC: nucleus pulposus progenitor cells
PI: propidium iodide
P/S: penicillin/streptomycin
PLGA: Poly (lactic-co-glycolic acid)
TEK: tyrosine kinase (Tie2)
TGFβ1: transforming growth factor beta-1
Tie2: angiopoietin-1 receptor
VEGF: vascular endothelial growth factor

## References

[1] Balagué F, Mannion AF, Pellisé F, Cedraschi C (2012). Non-specific low back pain. Lancet.

[2] Hoy D, March L, Brooks P, Blyth F, Woolf A, Bain C (2014). The global burden of low back pain: estimates from the Global Burden of Disease 2010 study. Ann Rheum Dis.

[3] Fourney DR, Andersson G, Arnold PM, Dettori J, Cahana A, Fehlings MG (2011). Chronic low back pain: a heterogeneous condition with challenges for an evidence-based approach. Spine (Phila Pa 1976).

[4] Urban JPG, Roberts S, Ralphs JR (2000). The nucleus of the intervertebral disc from development to degeneration. Am Zool.

[5] Agrawal A, Gajghate S, Smith H, Anderson DG, Albert TJ, Shapiro IM (2008). Cited2 modulates hypoxia-inducible factor-dependent expression of vascular endothelial growth factor in nucleus pulposus cells of the rat intervertebral disc. Arthritis Rheum.

[6] Masuda K, Oegema TR, An HS (2004). Growth factors and treatment of intervertebral disc degeneration. Spine (Phila Pa 1976).

[7] Hassett G, Hart DJ, Manek NJ, Doyle DV, Spector TD (2003). Risk factors for progression of lumbar spine disc degeneration: the Chingford Study. Arthritis Rheum.

[8] Johnson WE, Eisenstein SM, Roberts S (2001). Cell cluster formation in degenerate lumbar intervertebral discs is associated with increased disc cell proliferation. Connect Tissue Res.

[9] Roberts S, Evans H, Trivedi J, Menage J (2006). Histology and pathology of the human intervertebral disc. J Bone Joint Surg Am.

[10] Erwin WM, Islam D, Inman RD, Fehlings MG, Tsui FW (2011). Notochordal cells protect nucleus pulposus cells from degradation and apoptosis: implications for the mechanisms of intervertebral disc degeneration. Arthritis Res Ther.

[11] Yim RL, Lee JT, Bow CH, Meij B, Leung V, Cheung KM (2014). A systematic review of the safety and efficacy of mesenchymal stem cells for disc degeneration: insights and future directions for regenerative therapeutics. Stem Cells Dev.

[12] Sakai D, Andersson GB (2015). Stem cell therapy for intervertebral disc regeneration: obstacles and solutions. Nat Rev Rheumatol.

[13] Sakai D, Nakamura Y, Nakai T, Mishima T, Kato S, Grad S (2012). Exhaustion of nucleus pulposus progenitor cells with ageing and degeneration of the intervertebral disc. Nat Commun.

[14] Koblizek TI, Runttng AS, Stacker SA, Wilks AF, Risau W, Deutsch U (1997). Tie2 receptor expression and phosphorylation in cultured cells and mouse tissues. Eur J Biochem.

[15] Loughna S, Sato TN (2001). Angiopoietin and Tie signaling pathways in vascular development. Matrix Biol.

[16] Suri C, Jones PF, Patan S, Bartunkova S, Maisonpierre PC, Davis S (1996). Requisite role of angiopoietin-1, a ligand for the TIE2 receptor, during embryonic angiogenesis. Cell.

[17] Gantenbein B, Illien-Jünger S, Chan SC, Walser J, Haglund L, Ferguson SJ (2015). Organ culture bioreactors—platforms to study human intervertebral disc degeneration and regenerative therapy. Curr Stem Cell Res Ther.

[18] Barbero A, Grogan SP, Mainil-Varlet P, Martin I (2006). Expansion on specific substrates regulates the phenotype and differentiation capacity of human articular chondrocytes. J Cell Biochem.

[19] Stoyanov JV, Gantenbein-Ritter B, Bertolo A, Aebli N, Baur M, Alini M (2011). Role of hypoxia and growth and differentiation factor-5 on differentiation of human mesenchymal stem cells towards intervertebral nucleus pulposus-like cells. Eur Cell Mater.

[20] Yang X, Li X (2009). Nucleus pulposus tissue engineering: a brief review. Eur Spine J.

[21] Clarke LE, McConnell JC, Sherratt MJ, Derby B, Richardson SM, Hoyland JA (2014). Growth differentiation factor 6 and transforming growth factor-beta differentially mediate mesenchymal stem cell differentiation, composition and micromechanical properties of nucleus pulposus constructs. Arthritis Res Ther.

[22] Taupin P, Ray J, Fischer WH, Suhr ST, Hakansson K, Grubb A (2000). FGF-2-responsive neural stem cell proliferation requires CCg, a novel autocrine/paracrine cofactor. Neuron.

[23] Galas RJ, Liu JC (2014). Vascular endothelial growth factor does not accelerate endothelial differentiation of human mesenchymal stem cells. J Cell Physiol.

[24] Levenstein ME, Ludwig TE, Xu RH, Llanas RA, VanDenHeuvel-Kramer K, Manning D (2006). Basic fibroblast growth factor support of human embryonic stem cell self-renewal. Stem Cells.

[25] Tsai TL, Wang B, Squire MW, Guo LW, Li WJ (2015). Endothelial cells direct human mesenchymal stem cells for osteo- and chondro-lineage differentiation through endothelin-1 and AKT signaling. Stem Cell Res Ther.

[26] Feng G, Li L, Liu H, Song Y, Huang F, Tu C (2013). Hypoxia differentially regulates human nucleus pulposus and annulus fibrosus cell extracellular matrix production in 3D scaffolds. Osteoarthritis Cartilage.

[27] Mwale F, Ciobanu I, Giannitsios D, Roughley P, Steffen T, Antoniou J (2011). Effect of oxygen levels on proteoglycan synthesis by intervertebral disc cells. Spine (Phila Pa 1976).

[28] Gantenbein B, Calandriello E, Wuertz-Kozak K, Benneker LM, Keel MJ, Chan SC (2014). Activation of intervertebral disc cells by co-culture with notochordal cells, conditioned medium and hypoxia. BMC Musculoskelet Disord.

[29] Livak KJ, Schmittgen TD (2001). Analysis of relative gene expression data using real-time quantitative PCR and the 2(-Delta Delta C(T)) method. Methods.

[30] Oehme D, Goldschlager T, Ghosh P, Rosenfeld JV, Jenkin G (2015). Cell-based therapies used to treat lumbar degenerative disc disease: a systematic review of animal studies and human clinical trials. Stem Cells Int.

[31] Benneker LM, Andersson G, Iatridis JC, Sakai D, Härtl R, Ito K (2014). Cell therapy for intervertebral disc repair: advancing cell therapy from bench to clinics. Eur Cell Mater.

[32] Arkesteijn IT, Smolders LA, Spillekom S, Riemers FM, Potier E, Meij BP (2015). Effect of coculturing canine notochordal, nucleus pulposus and mesenchymal stromal cells for intervertebral disc regeneration. Arthritis Res Ther.

[33] Okuma M, Mochida J, Nishimura K, Sakabe K, Seiki K (2000). Reinsertion of stimulated nucleus pulposus cells retards intervertebral disc degeneration: an in vitro and in vivo experimental study. J Orthop Res.

[34] Sato M, Asazuma T, Ishihara M, Ishihara M, Kikuchi T, Kikuchi M (2003). An experimental study of the regeneration of the intervertebral disc with an allograft of cultured annulus fibrosus cells using a tissue-engineering method. Spine (Phila Pa 1976).

[35] Iwashina T, Mochida J, Sakai D, Yamamoto Y, Miyazaki T, Ando K (2006). Feasibility of using a human nucleus pulposus cell line as a cell source in cell transplantation therapy for intervertebral disc degeneration. Spine (Phila Pa 1976).

[36] Gorensek M, Jaksimović C, Kregar-Velikonja N, Gorensek M, Knezevic M, Jeras M (2004). Nucleus pulposus repair with cultured autologous elastic cartilage derived chondrocytes. Cell Mol Biol Lett.

[37] Henriksson H, Hagman H, Horn H, Lindahl L, Brisby B (2011). Investigation of different cell types and gel carriers for cell-based intervertebral disc therapy, in vitro and in vivo studies. J Tissue Eng Regen Med.

[38] Coric D, Pettine K, Sumich A, Boltes MO (2013). Prospective study of disc repair with allogeneic chondrocytes presented at the 2012 Joint Spine Section Meeting. J Neurosurg Spine.

[39] Sakai D, Mochida J, Yamamoto Y, Nomura T, Okuma M, Nishimura K (2003). Transplantation of mesenchymal stem cells embedded in Atelocollagen gel to the intervertebral disc: a potential therapeutic model for disc degeneration. Biomaterials.

[40] Jeong JH, Lee JH, Jin ES, Min JK, Jeon SR, Choi KH (2010). Regeneration of intervertebral discs in a rat disc degeneration model by implanted adipose-tissue-derived stromal cells. Acta Neurochir (Wien).

[41] Tam V, Rogers I, Chan D, Leung VY, Cheung KM (2014). A comparison of intravenous and intradiscal delivery of multipotential stem cells on the healing of injured intervertebral disk. J Orthop Res.

[42] Sheikh H, Zakharian K, De La Torre RP, Facek C, Vasquez A, Chaudhry GR (2009). In vivo intervertebral disc regeneration using stem cell-derived chondroprogenitors. J Neurosurg Spine.

[43] Wang H, Zhou Y, Huang B, Liu LT, Liu MH, Wang J (2014). Utilization of stem cells in alginate for nucleus pulposus tissue engineering. Tissue Eng Part A.

[44] Lee CR, Sakai D, Nakai T, Toyama K, Mochida J, Alini M (2007). A phenotypic comparison of intervertebral disc and articular cartilage cells in the rat. Eur Spine J.

[45] Shi R, Wang F, Hong X, Wang YT, Bao JP, Cai F, et al. The presence of stem cells in potential stem cell niches of the intervertebral disc region: an in vitro study on rats. Eur Spine J. 2015;24(11):2411-24. doi: 10.1007/s00586-015-4168-7. [Epub ahead of print]. 10.1007/s00586-015-4168-7 26228187

[46] Blanco JF, Graciani IF, Sanchez-Guijo FM, Muntión S, Hernandez-Campo P, Santamaria C (2010). Isolation and characterization of mesenchymal stromal cells from human degenerated nucleus pulposus: comparison with bone marrow mesenchymal stromal cells from the same subjects. Spine (Phila Pa 1976).

[47] Feng G, Yang X, Shang H, Marks IW, Shen FH, Katz A (2010). Multipotential differentiation of human anulus fibrosus cells: an in vitro study. J Bone Joint Surg Am.

[48] Sakai D, Grad S (2014). Advancing the cellular and molecular therapy for intervertebral disc disease. Adv Drug Deliv Rev.

[49] Maroudas A, Stockwell RA, Nachemson A, Urban J (1975). Factors involved in the nutrition of the human lumbar intervertebral disc: cellularity and diffusion of glucose in vitro. J Anat.

[50] Miyazaki T, Kobayashi S, Takeno K, Meir A, Urban J, Baba H (2009). A Phenotypic comparison of proteoglycan production of intervertebral disc cells isolated from rats, rabbits, and bovine tails; which animal model is most suitable to study tissue engineering and biological repair of human disc disorders?. Tissue Eng Part A.

[51] Showalter BL, Beckstein JC, Martin JT, Beattie EE, Espinoza Orías AA, Schaer TP (2012). Comparison of animal discs used in disc research to human lumbar disc: torsion mechanics and collagen content. Spine (Phila Pa 1976).

[52] Kim YH, Yoon DS, Kim HO, Lee JW (2012). Characterization of different subpopulations from bone marrow-derived mesenchymal stromal cells by alkaline phosphatase expression. Stem Cells Dev.

[53] Sun HJ, Bahk YY, Choi YR, Shim JH, Han SH, Lee JW (2006). A proteomic analysis during serial subculture and osteogenic differentiation of human mesenchymal stem cell. J Orthop Res.

[54] Prockop DJ, Sekiya I, Colter DC (2001). Isolation and characterization of rapidly self-renewing stem cells from cultures of human marrow stromal cells. Cytotherapy.

[55] Seghezzi G, Patel S, Ren CJ, Gualandris A, Pintucci G, Robbins ES (1998). Fibroblast growth factor-2 (FGF-2) induces vascular endothelial growth factor (VEGF) expression in the endothelial cells of forming capillaries: an autocrine mechanism contributing to angiogenesis. J Cell Biol.

[56] Lotz S, Goderie S, Tokas N, Hirsch SE, Ahmad F, Corneo B (2013). Sustained levels of FGF2 maintain undifferentiated stem cell cultures with biweekly feeding. PLoS One.

[57] Mohyeldin A, Garzón-Muvdi T, Quiñones-Hinojosa A (2010). Oxygen in stem cell biology: a critical component of the stem cell niche. Cell Stem Cell.

[58] Yang Z, Sui L, Toh WS, Lee EH, Cao T (2009). Stage-dependent effect of TGF-beta1 on chondrogenic differentiation of human embryonic stem cells. Stem Cells Dev.

