# Correction to: Angiopoietin-1 receptor Tie2 distinguishes multipotent differentiation capability in bovine coccygeal nucleus pulposus cells

**DOI:** 10.1186/s13287-019-1150-z

**Published:** 2019-01-31

**Authors:** Adel Tekari, Samantha C. W. Chan, Daisuke Sakai, Sibylle Grad, Benjamin Gantenbein

**Affiliations:** 1 Tissue and Organ Mechanobiology, Institute for Surgical Technology & Biomechanics, Medical Faculty, University of Bern, Bern, Switzerland; 2 Biointerfaces, EMPA, Swiss Federal Laboratories for Materials Science and Technology, St Gallen, Switzerland; 3 Department for Orthopaedic Surgery, Tokai University School of Medicine, Isehara, Kanagawa Japan; 4 AO Research Institute Davos, Davos, Switzerland; 5 AO Spine Research Network, AO Spine International, Davos, Switzerland

**Correction to: Stem Cell Res Ther (2016) 7:75**

**https://doi.org/10.1186/s13287-016-0337-9**

Following publication of the original article in Stem Cell Research & Therapy [1], we would like to alert the reader that the immune-histological sections shown in Fig. 2 bottom line are mistakenly the images from an experiment using a different Tie2+ antibody than originally reported in the manuscript (i.e. R&D, anti-human Tie2 labeled APC, cat.No:FAB3131A, clone:83715, mouse IgG) for the florescence associated cell sorting (FACS). This antibody has been previously tested in the group of Prof. Dr. Daisuke Sakai and was performed by Ms Tomoko Nakai, Tokai University. This antibody, however, was not found to be specific for bovine Tie2+ cells. The immune-histology procedure was correctly described using the PG antibody from Millipore. However, the pictures presented in Fig. 2 in the last raw in the article of Tekari *et al.* [1] are not from the same experiment using the Tie2 antibody from Bioss, inc. clone bs-1300R, Bioss Antibodies, Woburn, MA, USA, as the publication reported.

**Fig. 2:**
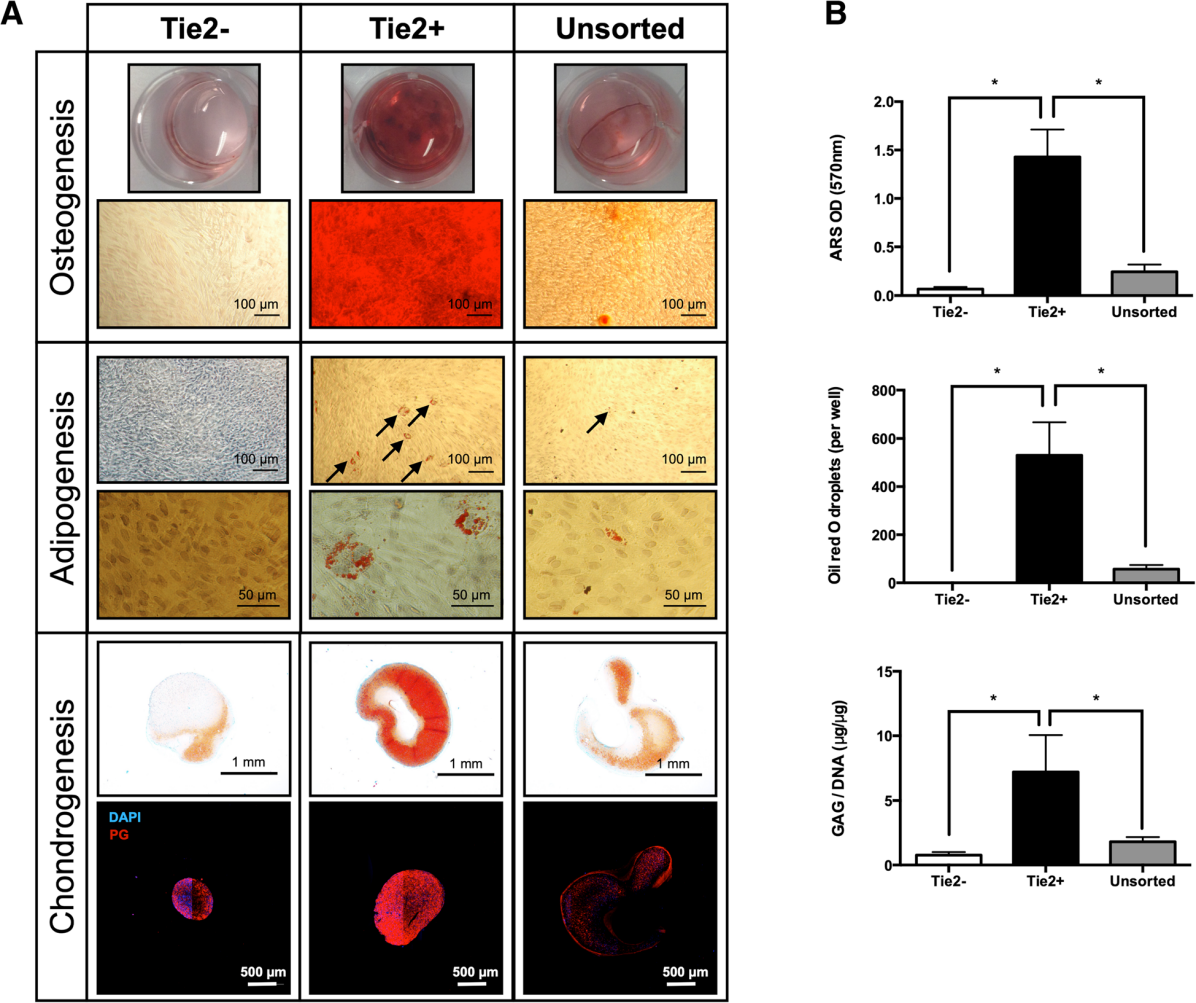
Osteogenic, adipogenic and chondrogenic differentiation assays. **a** The differentiation assays were performed in Tie2-, Tie2+ (i.e. NPPC) cells after sorting and a mixed cell population (unsorted NPC). Top panel represents the macroscopic and microscopic images of osteogenesis (Alizarin red staining). Middle panel represents the adipogenic differentiation (Oil red O staining), and arrows highlight the formation of fat droplets. Lower panel represents the chondrogenic differentiation: Safranin-O staining and proteoglycans (PG, red) immunohistochemistry counterstained with DAPI (4',6-diamidino-2-phenylindole, blue). Results of one representative experiment of at least 3 repeats are shown. Scale bars are indicated on the images. **b** Quantification of Alizarin red staining (ARS), Oil red O fat droplets positive cells and GAG/DNA content. Individual cell populations were cross-compared to determine significance with *p < 0.05. Bars represent mean ± SD (N=5)

We have now fixed this issue by providing a new Fig. 2 using the reported Tie2 primary antibody from Bioss and the secondary antibody labeled with Alexa 488 (cat# A-11008, Molecular Probes, Life Technologies, Zug, Switzerland) for FACS sorting.

The corrected Material and Methods Section for this part on page 4 of the original article should read:

“Immunohistochemical staining for proteoglycans was performed by incubation of the sections with a monoclonal mouse anti-human proteoglycan antibody (10 μg/ml, clone EFG-4; Millipore, Billerica, MA, USA, diluted 1:50) overnight at 4 °C after permeabilization with 100 % methanol for 2 min, rehydration and blocking with 10 % FBS in phosphate buffered saline (PBS) for 1 hour. Secondary antibody was then added after stringent washing for 1 hour on the next day, which was a goat anti-mouse antibody (Alexa Fluor 555 goat anti-mouse SFX-Kit IgG A-31621, Invitrogen, Fisher-Scientific, Basel, 1:200 diluted). Finally, slides were mounted in DAPI containing embedding medium (Fluoroshield™ cat# ab104139, abcam plc, Cambridge, UK). Images were then taken with a confocal laser scanning microscope at a 10x magnification and using 4x4 tile imaging (cLSM710, Carl Zeiss, Jena, Germany).”
